# A study of docetaxel and irinotecan in children and young adults with recurrent or refractory Ewing sarcoma family of tumors

**DOI:** 10.1186/1471-2407-14-622

**Published:** 2014-08-28

**Authors:** Jong Hyung Yoon, Mi Mi Kwon, Hyeon Jin Park, Seog Yun Park, Kun Young Lim, Jungnam Joo, Byung-Kiu Park

**Affiliations:** Center for Pediatric Oncology, National Cancer Center, 323 Ilsan-ro, Ilsandong-gu, Goyang-si, Gyeonggi-do 410-769 Korea; Department of Pathology, National Cancer Center, Goyang, Korea; Department of Radiology, National Cancer Center, Goyang, Korea; Biometric Research Branch, National Cancer Center, Goyang, Korea

**Keywords:** Docetaxel, Irinotecan, Recurrent, Refractory, Ewing sarcoma family of tumors

## Abstract

**Background:**

Patients with Ewing sarcoma family of tumors (ESFT) who are resistant even to salvage chemotherapy, have dismal prognoses and few therapeutic options. Because the docetaxel/irinotecan (DI) combination has not been previously evaluated in ESFT, we prospectively evaluated its use in patients with recurrent or refractory ESFT.

**Methods:**

Patients aged <30 years with ESFT, who failed ≥ third-line therapy, were eligible. They received docetaxel 100 mg/m^2^ intravenously on day 1, and irinotecan 80 mg/m^2^ on days 1 and 8, of a 21-day cycle up to 15 cycles or until disease progressed. The primary objective was objective response rate (ORR); secondary objectives were progression-free survival (PFS) and safety.

**Results:**

We enrolled nine patients (median age: 13 years); four were male. Two patients had recurrent disease and seven had progressive disease. This group had undergone a median of four prior chemotherapy regimens (range: 3-6), and received a total of 51 DI cycles (median: three cycles/per person; range: 1-15 cycles). The nine patients showed one complete response (CR), two partial responses (PRs), one stable disease, and five progressive diseases, for an ORR (CR + PR) of 3/9 (33.3%). Two patients with PR achieved CR with subsequent surgery. Overall median PFS was 2.2 months (range: 0.5-16.9 months). All nine patients had grade 4 neutropenia (100%); grade 3 diarrhea or grade 2/3 neuropathy each occurred in two patients (22%). All toxicities were manageable without serious morbidities or treatment-related mortality.

**Conclusions:**

The DI combination may be effective and tolerable for patients with heavily pre-treated ESFT.

**Trial registration:**

NCT01380275. Registered June 21, 2011.

## Background

The Ewing sarcoma family of tumors (ESFT) is the second most common primary bone malignancy in children and young adults [1]. It is a group of small, blue round cell neoplasms of neuroectodermal origin, which includes classical Ewing sarcoma, primitive neuroectodermal tumors, and Askin tumors of the chest wall [2]. With multimodality treatment, the 5-year survival rate for locoregional ESFT is 60-75% depending on various series [3, 4]. However, the 5-year survival rate for metastatic disease at diagnosis is less than 30% [5], and for recurrent or refractory disease it falls even below 20% [6].

As no standard salvage regimen exists for recurrent or refractory ESFT, various regimens have been tried as second-line agents, such as topotecan plus cyclophosphamide (TC), ifosfamide, carboplatin plus etoposide (ICE), temozolomide plus irinotecan (TI), and gemcitabine plus docetaxel (GD), yielding response rates varying between 29% and 66% [7–10]. However, many patients fail to respond to these second-line agents, and chemotherapeutic options are further limited when these agents also fail. Furthermore, despite strenuous efforts, the survival rate has not increased in patients with recurrent or refractory ESFT over the last several decades, which highlights the need for more effective regimens.

Docetaxel is a semisynthetic taxane analog, which acts as a mitotic spindle poison by promoting microtubule assembly but inhibiting tubulin depolymerization, thus disrupting cell division [11]. It has shown activity in patients with many types of solid tumors, including ESFT [11]. Irinotecan is a camptothecin prodrug that is metabolized by carboxylesterase to an active metabolite, SN-38 [12]. SN-38 is a potent inhibitor of topoisomerase I, a critical enzyme in DNA replication and transcription. Irinotecan has also shown a significant activity in ESFT [12]. Docetaxel and irinotecan (DI) have demonstrated additive and synergistic activities *in vitro* and *in vivo* [13, 14]. Additionally, they have different biologic targets and mechanisms of resistance from first-line and many second-line agents for ESFT. Thus, DI may provide a suitable option in ESFT that shows resistance to other regimens. However, the combination has not been evaluated in ESFT to date.

Herein, we prospectively evaluated the efficacy and toxicity profile of the DI combination in children and young adults with recurrent or refractory ESFT. In this report, we demonstrated that the combination is effective and tolerable in our heavily pre-treated cohort.

## Methods

### Patient eligibility

We recruited patients with histologically confirmed ESFT who received DI chemotherapy at the National Cancer Center, Korea, following relapse or progression with ≥ third-line therapy. This study was originally designed as a single-arm phase II trial to estimate the complete response (CR) plus partial response (PR) rates, with precision. Twenty-eight patients were required to estimate the expected response rate of 25% with a 95% confidence interval (CI) of ±15%. Assuming 20% follow-up loss, the intended sample size required was 34. However, because of slow recruitment caused by financial difficulties on participants’ side, the recruitment was stopped prematurely after enrolling nine patients. Patients aged <30 years with recurrent or refractory ESFT that was inoperable at study entry, were eligible. Prior paclitaxel or topotecan use was permitted, but prior therapy with docetaxel or irinotecan at any time was not allowed. Patients with Karnofsky (age >10 years) or Lansky (age ≤10 years) scores ≥50, and measurable disease were eligible. ESFT was confirmed by a single pathologist. For all nine tumor specimens, reverse transcription-polymerase chain reaction and/or fluorescence *in situ* hybridization were conducted, revealing *EWS/Fli-1* translocation. Patients who had completed at least one cycle of DI were evaluated for chemotherapy response according to Response Evaluation Criteria in Solid Tumors (RECIST). At the beginning of each cycle, patients were required to have adequate hematologic values (absolute neutrophil count [ANC] ≥750/μL and platelet count ≥75,000/μL), renal function (serum creatinine <1.5× upper normal limit for age or creatinine clearance ≥60 mL/min/1.73 m^2^), and liver function (total bilirubin ≤1.5 mg/dL and AST/ALT levels <2.5× upper normal limits), and no other non-hematologic toxicity of grade 2 or worse. Patients could not receive other anti-cancer or investigational agents during the study period or within 4 weeks prior to study entry. Patients who had been taking anticonvulsants that might affect the metabolism of DI, who were pregnant or lactating, had psychiatric disorders, uncontrolled infections, pre-existing neuropathy grade ≥2, or brain metastases were also excluded. Data were collected on gender, age, sites of primary tumor and metastasis, previous surgery or radiotherapy, previous chemotherapy regimens, date of the last chemotherapy, date of DI commencement, performance status, number of cycles, best response, number of admissions for toxicities and type of toxicities, date of progression, and date of death or the last follow-up.

All parents/guardians and/or patients gave informed consent; the study was approved by the Institutional Review Board of the National Cancer Center, Korea (IRB number: NCCCTS-08-322) and complied with local laws and regulations, and the Declaration of Helsinki.

### Chemotherapeutic regimen

Docetaxel (Taxotere®; Sanofi-Aventis, Bridgewater, NJ, USA) was administered as a 60-min intravenous (IV) infusion at a dose of 100 mg/m^2^ followed by the administration of 80 mg/m^2^ irinotecan (Irinotecan®; Boryung Pharmaceutical Co., Ltd, Seoul, Korea) IV over 90 min. Docetaxel was given on day 1, and irinotecan on days 1 and 8 of a 21-day cycle. The cycles continued until progression of disease, the occurrence of unacceptable toxicities, or patient withdrawal. The planned maximum number of cycles was 12; however, two to three additional cycles were given at the discretion of physicians when at least partial response was maintained at the end of 12 cycles.

Patients were premedicated with antiemetic agents and dexamethasone for a total of three doses. Atropine and loperamide was used to treat early-onset (within 12 h after irinotecan infusion) and delayed-onset diarrhea, respectively, according to the reported guideline [15].

Irinotecan treatment on day 8 was delayed to day 10 if grade ≥2 non-hematological toxicities, such as diarrhea, occurred on the day when the dose was due. It was omitted if grade ≥2 non-hematological toxicities continued to day 10. If diarrhea of grade ≥3 persisted despite atropine or loperamide use, the irinotecan dose in the next cycle was reduced by 20%. Subsequent doses were further reduced by 20% for recurrent toxicities.

Doses of docetaxel in the subsequent cycles were reduced by 20% if grade ≥2 neurotoxicity or recurrent fluid retention, or any grade ≥3 non-hematologic toxicities occurred, including hepatotoxicity, myalgia, cardiac events, or hypersensitivity. However, doses were not reduced for grade 3 oral mucositis or infection. Subsequent doses were further reduced by 20% for recurrent toxicities. Doses of both docetaxel and irinotecan in subsequent cycles were reduced by 20% if septicemia occurred. Granulocyte colony stimulating factor (G-CSF) injections were initiated if absolute neutrophil count (ANC) fell below 500/μL and continued until ANC ≥1,000/μL. Dose re-escalation after a dose reduction was not permitted for either docetaxel or irinotecan. DI therapy was discontinued if grade 4 non-hematological toxicities occurred.

### Assessment of tumor response and toxicities

Tumor response was assessed by magnetic resonance imaging or computed tomography (CT) scanning, according to RECIST criteria version 1.1 [16], at baseline and then every two or three cycles. When clinically indicated, images were obtained earlier. We defined CR as a complete regression of all apparent tumor masses and PR as >30% decrease in the longest diameter of primary and/or metastatic tumors with the absence of new lesions. Progressive disease (PD) was defined as >20% increase in the longest diameter of primary or metastatic tumors or the appearance of new lesions. Stable disease (SD) was defined as the absence of CR, PR, or PD. Either CR or PR was regarded as an objective response (OR). When a patient was deemed to have a CR, PR, or SD, tumor measurement was repeated 4-6 weeks later to confirm the response. Images were reviewed by a single radiologist who was blinded to clinical information. Complete blood counts were obtained on days 1 and 8 of each cycle, and serum chemistry results were obtained on day 1.

Adverse events and laboratory variables were assessed using the National Cancer Institute’s Common Terminology Criteria for Adverse Events, version 4.0 (http://evs.nci.nih.gov/).

### Statistical analysis

The primary objective was the best OR rate (ORR), and the secondary objectives were progression-free survival (PFS) and toxicity profile.

Two parameters were used to evaluate efficacy, best response over the entire duration of treatment, and PFS (time between the first day of DI therapy and the date of disease progression, death from any cause, or the last follow-up). Differences in response rate were tested using the Fisher’s exact test because of the small sample size. The Kaplan-Meier method was used to estimate median PFS; Greenwood’s formula was used to find its 95% CI. The differences in survival were tested using the log rank test. Two-sided *P* values were reported for all statistical analyses. *P* <0.05 was considered significant. Statistical analyses were performed using STATA 12.0 for Windows 9 (StataCorp, College Station, TX, USA).

## Results

### Patient characteristics

Between July 2008 and January 2013, a total of 10 consecutive ESFT patients with relapsed or progressed disease were treated at the Center for Pediatric Oncology of the National Cancer Center, Korea. Of the 10 patients, one was excluded because of an ineligible performance score. The remaining nine all completed at least one cycle and were analyzed for their DI responses and toxicities. Patient characteristics at enrollment and their prior treatments are shown in Table 1. Four (44%) of the nine patients were male. Median age at study entry was 13 years (range: 5-21 years). The most common primary site (56%) was the pelvic bone and/or pelvic cavity, including one at the lumbosacral spine with adjacent epidural sac and presacral space. Five patients (56%) had metastatic diseases at initial diagnosis. Eight (89%) had metastases at enrollment, most commonly at the lungs (8/8, 100%) followed by the pleura (5/8, 63%). The patients had undergone a median of four previous chemotherapeutic regimens (range: 3-6) (Table 1). Seven patients had undergone surgery for primary tumors, but only two were left with negative resection margins; two had inoperable primary sites. Three of the five patients with lung metastases at initial diagnosis underwent wedge resections of lung nodules, leaving negative resection margins of 1-2 mm. Eight of the nine patients had received radiation therapy for primary and/or metastatic sites, and five had undergone high-dose chemotherapy and autologous stem cell transplantation before enrollment.

**Table 1:** **Patient characteristics and their previous therapies at enrollment (n = 9)**

UPN	Primary sites	Metastatic sites at initial diagnosis	Involved sites at enrollment	No. of prior chemotherapy regimens	Prior surgery	Prior radiation therapy	HDC/ASCT
1	Lt ilium	Lungs, pleura, LS spines, Rt femur, and tibia	Lungs, pleura	6	Resection of pelvic mass (NM) and lung nodules (NM)	Yes	Yes
2	Anterior mediastinum and chest wall	Lungs	Lungs, pleura	4	Resection of mediastinal and chest wall mass (PM), and lung nodules (NM)	Yes	Yes
3	Pubis and pelvic cavity	None	PS, lungs, LS spines with epidural sac	4	Resection of pelvic mass (PM)	Yes	No
4	Pubis, Lt ischium, and pelvic cavity	None	PS	5	No	No	No
5	Lt chest wall and ribs	Lungs, pleura	PS, lungs, pleura, brain, liver, kidney	6	Resection of chest wall mass (PM)	Yes	No
6	Abdomino-pelvic cavity	Lungs	Lungs, pleura, and Lt. interlobar L/Ns	3	Resection of abdomino-pelvic mass (PM)	Yes	Yes
7	Lt paraspinal muscle at LS level	Lungs, Lt common iliac L/Ns	PS, lungs, L spines, Lt ilium, and Lt common iliac L/Ns	3	Resection of paraspinal mass (PM) and lung nodules (NM)	Yes	No
8	LS spines with epidural sac and presacral space	None	PS, lungs	3	No	Yes	Yes
9	Lt Humerus	None	Lungs, pleura	3	Resection of humeral mass (NM)	Yes	Yes

ASCT, autologous stem cell transplantation; HDC, high-dose chemotherapy; L, lumbar; L/Ns, lymph nodes; LS, lumbosacral; Lt, left; NM, negative margin; PM, positive margin; PS, primary site; Rt, right; UPN, unique patient number.

Previous chemotherapeutic regimens delivered are summarized in Table 2. Various chemotherapeutic or biological agents had been used in the patients’ chemotherapy regimens. Vincristine, ifosfamide, doxorubicin, and etoposide with or without vincristine, actinomycin-D, and ifosfamide had been used in five (56%) patients as a first-line agent. As salvage regimens, TC with or without etoposide, and/or carboplatin had been administered in 10 (36%) of total 28 regimens, and ICE with or without vincristine in seven regimens. Actinomycin-D, cytarabine, paclitaxel, cisplatin, and zoledronate had also been used in various combinations. Unique patient number (UPN) 1 had received an insulin-like growth factor-1 receptor antibody after disease progression without effect. As conditioning regimens delivered before stem cell transplantation, busulfan/melphalan had been used in two patients and melphalan/etoposide- based regimens in the others (Table 2).

**Table 2:** **Previous chemotherapeutic regimens administered before study enrollment**

Regimens	Number (%)
**First-line chemotherapy**	**n = 9**
VIDE with or without VAI regimen	5 (56)
VDC/IE regimen	3 (33)
ICE	1 (11)
**Salvage chemo and/or biotherapy**	**n = 28**
TC with or without etoposide and/or carboplatin	10 (36)
ICE or VICE	7 (25)
VAC	2 (7)
Others^*^	9 (32)
**Conditioning regimens for ASCT**	**n = 5**
BuMel	2 (40)
MET	1 (20)
METBI	1 (20)
MEC	1 (20)

^*^Cytarabine, paclitaxel, cisplatin, and zoledronate were used in various combinations.

Abbreviations: ASCT, autologous stem cell transplantation; BuMel, busulfan/melphalan; ICE, ifosfamide/carboplatin/etoposide; MEC, melphalan/etoposide/carboplatin; MET, melphalan/etoposide/thiotepa; METBI, melphalan/etoposide/total body irradiation; TC, topotecan/cyclophosphamide; VAC, vincristine/actinomycin-D/cyclophosphamide; VAI, vincristine/actinomycin-D/ifosfamide; VDC/IE, vincristine/doxorubicin/cyclophosphamide alternating with ifosfamide/etoposide; VICE, vincristine/ifosfamide/carboplatin/etoposide; VIDE, vincristine/ifosfamide/doxorubicin/etoposide.

### Tumor response

**Figure 1:**
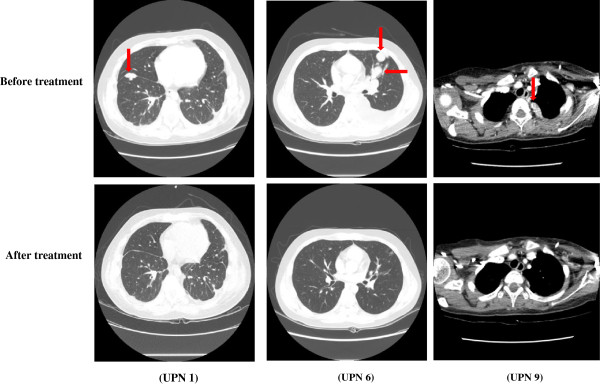
**Computed tomography chest scans of three patients, showing response to docetaxel and irinotecan (DI) combination.** No lung lesion was visible (complete response) after five cycles of DI in unique patient number (UPN) 1. UPNs 6 and 9 showed decreases in tumor (arrow) measurements of 40% after five cycles and 50% after three cycles, respectively, indicating partial responses by Response Evaluation Criteria in Solid Tumors (RECIST) version 1.1.

Reasons for DI therapy, number of DI cycles administered, DI response, and treatment outcomes are described in Table 3. At the time of study entry, seven patients had progressive/refractory disease; one suffered from a second relapse; and one experienced a third relapse. The patients received a median of three DI cycles (range: 1-15). Two patients received only one cycle each, owing to overt disease progression. Three experienced progression after two or three cycles. One (UPN 8) showed SD after two cycles, but PD after four cycles, and the SD was not confirmed in repeat imaging. The remaining three patients reported responses. One (UPN 1) had a CR (Figure 1) after five cycles and received nine more cycles (14 cycles in all). Unfortunately, he developed a lung relapse 2 months after completing the DI regimen. Another two patients (UPNs 6 and 9) had PRs (Figure 1) after five and three cycles, respectively, which rendered their lung lesions resectable, and underwent excision of pulmonary metastases after eight and three cycles, respectively, which resulted in CR. Pathology revealed metastatic ESFT. After surgery, UPN 6 continued to have DI and received 15 cycles in total, but developed lung relapse 4 months post-DI therapy. UPN 9 received nine cycles in total, and remained in CR while on therapy (as of analysis on July 31, 2013). No response was observed in the five patients who had primary refractory disease (no previous CR), while three of the four patients with secondary refractory disease (presence of previous CR with subsequent refractoriness) or relapse responded to DI (*P* = 0.048 with Fisher’s exact test). Moreover, all three responders had isolated lung and pleural lesions as metastatic sites on enrollment (Table 1).

**Table 3:** **Reason for DI therapy, number of DI cycles, DI response, and patient outcome (n = 9)**

UPN	Reason for DI therapy	No. of DI cycles administered	Study progress	Best response	Outcome	PFS (months)	Follow-up period (months)
1	SPD	14	Completed	CR	DOD	12.4	17.1
2	2^nd^ relapse	3	Stopped	PD	DOD	2.2	24.6
3	PPD	1	Stopped	PD	DOD	0.5	1.5
4	PPD	2	Stopped	PD	DOD	1.4	1.9
5	PPD	1	Stopped	PD	DOD	0.5	1
6	SPD	15	Completed	PR*	AWD	16.9	41.3
7	PPD	2	Stopped	PD	DOD	1.4	6.2
8	PPD	4	Stopped	SD^†^	DOD	3.1	10.1
9	3^rd^ relapse	9	Ongoing	PR*	NED	6.4	6.4

*Two patients achieved PR with DI chemotherapy, followed by CR with subsequent resection of pulmonary metastases.

^†^SD was not confirmed in repeat imaging.

*Abbreviations*: AWD, alive with disease; CR, complete response; DI, docetaxel and irinotecan; DOD, died of disease; NED, no evidence of disease; PD, progressive disease; PFS, progression-free survival; PPD, primary progressive disease; PR, partial response; SD, stable disease; SPD, secondary progressive disease; UPN, unique patient number.

In the entire cohort, 1 CR and 2 PRs were obtained, for an ORR of 33% (3/9) (95% CI, 7.5-70.1).

### Survival results

**Figure 2:**
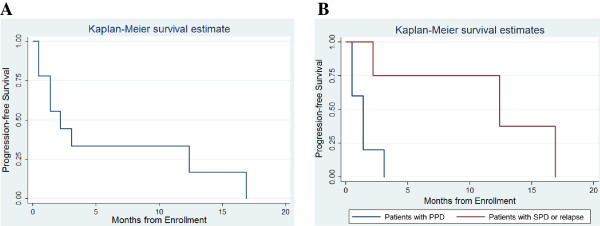
**Progression-free survival (PFS). (A)** Kaplan-Meier estimation of PFS of all nine patients; and **(B)** difference in median PFS between five patients with primary progressive disease (PPD) and four with secondary progressive disease (SPD) or relapse (*P* = 0.018).

At the time of our analysis, seven patients (78%) had died of disease progression, and two (22%) were alive (Table 3). Metastatic tumors in the two surviving patients shrunk enough for them to undergo metastatectomy with DI therapy, leaving negative resection margins of 5 mm (UPN 6) and 7 mm (UPN 9). The median follow-up period was 6.4 months (range: 1.0-41.3 months). The median PFS of the cohort was 2.2 months (range: 0.5-16.9 months), and the estimated 6- and 12-month PFS rates were 33.3 ± 15.7% (mean ± SD) for each (Figure 2A). Outcome was better in patients with secondary PD or relapse compared with those with primary PD (median PFS: 1.4 versus 12.4 months; *P* = 0.018 [log-rank test]) (Figure 2B).

### Toxicities

All patients were evaluable for toxicities (Table 4). The cohort received a total of 51 DI cycles.

**Table 4:** **Toxicity profile** ^*^ **in nine eligible patients who received a total of 51 cycles**

	Grade 2		Grade 3		Grade 4		Grade 3/4
	No. of events (%)	No. of patients (%)	No. of events (%)	No. of patients (%)	No. of events (%)	No. of patients (%)	No. of patients (%)
**Hematologic**							
Anemia	27 (53)	6 (67)	12 (24)	7 (78)	5 (10)	2 (22)	7 (78)
Thrombocytopenia	5 (10)	4 (44)	7 (14)	3 (33)	4 (8)	3 (33)	6 (67)
Leucopenia	-	-	2 (4)	1 (11)	49 (96)	8 (89)	9 (100)
Neutropenia	-	-	-	-	51 (100)	9 (100)	9 (100)
Neutropenic fever	-	-	6 (12)	5 (56)	-	-	5 (56)
Eye infection	-	-	1 (2)	1 (11)	-	-	1 (11)
Nail infection	1 (2)	1 (11)	-	-	-	-	-
Anorectal infection	-	-	1 (2)	1 (11)	-	-	1 (11)
**Non-hematologic**							
Headache	1 (2)	1 (11)					-
Dysgeusia	1 (2)	1 (11)	-	-	-	-	-
Oral mucositis	5 (10)	4 (44)	2 (4)	1 (11)	-	-	1 (11)
Nausea	4 (8)	2 (22)	-	-	-	-	-
Vomiting	9 (18)	6 (67)	-	-	-	-	-
Abdominal pain	6 (12)	3 (33)	-	-	-	-	-
Diarrhea	17 (33)	5 (56)	2 (4)	2 (22)	-	-	2 (22)
Chest discomfort	1 (2)	1 (11)	-	-	-	-	-
Pleural effusion	3 (6)	2 (22)	-	-	-	-	-
Pericardial effusion	1 (2)	1 (11)	-	-	-	-	-
Edema	1 (2)	1 (11)	-	-	-	-	-
Peripheral neuropathy	6 (12)	2 (22)	9 (18)	1 (11)	-	-	1 (11)
Tremor	1 (2)	1 (11)	-	-	-	-	-
Muscle weakness	1 (2)	1 (11)	14 (27)	1 (11)	-	-	1 (11)

^*^If a patient suffered from toxicity of more than one grade, each grade was counted separately.

*Abbreviations*: -, none.

Principal toxic effects were hematologic and gastrointestinal. All patients experienced grade 4 neutropenia after each cycle, which lasted for a median of 5 days per cycle (range: 2-11 days). Six episodes (6/51, 12%) of grade 3 neutropenic fever occurred in five patients (5/9, 56%), but no single episode of septicemia occurred. One patient (UPN 6) suffered from an orbital cellulitis, and another patient (UPN 5) developed an anal abscess with growth of *K. pneumonia*. Both of these infections were controlled by IV antibiotics without further complications. Grade 3/4 anemia and thrombocytopenia occurred in 17 (34%) and 11 (22%) cycles, respectively, without provoking any significant cardiopulmonary symptoms or bleeding. As for non-hematologic toxicities, no nausea or vomiting of grade ≥3 occurred. Although grade 2 diarrhea occurred in 17 cycles (33%), grade 3 diarrhea occurred only in two cycles (4%). Grade 2 fluid retention, including pleural effusion, pericardial effusion, or edema, which occurred after the fifth and ninth cycle in two patients (UPNs 1 and 6, respectively), was managed with dexamethasone and diuretics, resulting in partial resolution with no further aggravation. Two patients (UPNs 1 and 6) developed grade 2/3 peripheral neuropathy in 15 cycles (29%), which was controlled with gabapentin with or without amitriptyline.

Only one chemotherapy cycle was delayed longer than 2 weeks, in UPN 6 owing to orbital cellulitis. Dose reduction of docetaxel by 20% was required in two patients because of grade 2/3 neurotoxicity and grade 2 fluid retention. Dose reduction of irinotecan by 20% was required in two patients because of grade 3 diarrhea. Although five patients were hospitalized, for a total of eight times, for neutropenic fever (6/8, 75%), diarrhea, or oral mucositis, they fully recovered from these events. No treatment-related mortality occurred during the study period.

## Discussion

This study, to the best of our knowledge, is the first to evaluate the efficacy and toxicity of docetaxel in combination with irinotecan in ESFT. The reported 33% ORR is notable considering that all patients in our study were heavily pre-treated and had received three or more chemotherapy regimens. Previous studies of recurrent and/or refractory ESFT have documented that the ORR to various salvage regimens has a range of 29-66%. The reported response rate in this study is comparable to or lower than those achieved by TC (33-35%), ICE (48%), TI (29-63%), or GD (66%) [7–10, 17, 18]. However, these regimens have been used mostly in the second- or third-line settings, whereas our DI chemotherapy was used as a ≥ fourth-line agent. In addition, ICE and TC had been already attempted in seven or eight patients before study entry, suggesting the usefulness of DI even in ESFT cases that fail commonly used salvage regimens. In one report, four of the six patients responded to GD; however, these patients reportedly had undergone only up to three previous regimens [10]. Although a high 63% response rate was reported in 19 patients with recurrent/progressive ESFT treated with TI [17], it was conducted mostly in the second-line setting. In comparison, in another study of the efficacy of TI in 14 patients with recurrent/progressive ESFT who had been more aggressively pre-treated, ORR declined to 29% [9]; the clinical status of these 14 patients (when TI was started) was first relapse in six patients and second or further relapse in eight patients, which indicates that our cohort was even more heavily pre-treated. In fact, our cohort was characterized by one of the highest representation of heavily pre-treated patients, which is associated with likely resistance to multiple chemotherapeutic agents.

Phase II trials of DI, using 1- or 3- week dosing schedules, have been conducted in various adult solid tumors, including esophageal, gastric, and non-small cell lung cancers [19–21]. A study of adult patients with advanced gastric cancer showed a 60% ORR, with the starting dose of irinotecan 160 mg/m^2^ and docetaxel 65 mg/m^2^ every 3 weeks [20]. However, dose reductions (irinotecan to 120 mg/m^2^ and docetaxel to 50 mg/m^2^) because of toxicity reduced the response rate to 10.3%, indicating that the higher dose is critical for response rate. A phase I study has shown that, with filgrastim support, docetaxel dose at 100-185 mg/m^2^ every 3 weeks can be used for phase II trials of children with refractory solid tumors [22]. We thus used an intensified irinotecan dose of 160 mg/m^2^ (80 mg/m^2^ on days 1 and 8 per cycle) and a docetaxel dose of 100 mg/m^2^, considering an otherwise dismal outcome of our series. Although docetaxel administered on a weekly basis is known to be less myelosuppressive [23], we administered docetaxel every 3 weeks to reduce cost without causing decreased efficacy [24]. Similarly, weekly irinotecan dosing was used because of a limited free drug supply, although protracted dosing schedules are commonly used in pediatric trials [9, 18].

Although the three patients who achieved CR with DI chemotherapy with or without surgery reflect encouraging results, considering the heavily pretreated status of our cohort, the duration of remission did not last long (median CR duration: 8.9 months; range: 4.4-10.6 months). Similarly, a very recent report on the use of vincristine, irinotecan, and temozolomide mostly as a second-line therapy in patients with relapsed or refractory Ewing sarcoma showed that despite the high ORR of 54%, only 22% of patients were alive after a median follow-up of 10.3 months [18]. Hence, approaches to maintain a durable remission, such as maintenance therapy, are required. To this end, biological agents might be an option because less toxic agents are desirable in the heavily pre-treated setting.

Although the small cohort size precluded analysis of factors related to DI response, two important findings warrant attention. First, all patients who responded to DI had isolated pulmonary lesions at protocol entry. In accordance with this finding, post-recurrence survival advantage has been shown in ESFT patients with isolated pulmonary recurrences [25]. Of the three DI-responsive patients, two patients with PR survived while the patient with CR did not. The two surviving patients underwent metastatectomy for their shrunken pulmonary metastases with DI therapy, but the non-surviving patient did not do so before complete resolution of his pulmonary disease because he had a previous pulmonary metastatectomy. Second, no patients with primary PD responded, which implies inherent chemoresistance. In addition to these findings, although the small sample size precluded statistical analysis of the possible relationship between patients’ number of previous chemotherapeutic regimens versus DI response or PFS, the subgroup with four or more previous regimens reported 1/5 (20%) response and median PFS of 1.4 months (range: 0.5-12.4 months), compared with the subgroup with less than four previous regimens who reported 2/4 (50%) response and median PFS of 4.8 months (range: 1.4-16.9 months).

With regard to hematologic toxicities, grade 3/4 leucopenia or neutropenia occurred in all patients, and grade 3/4 anemia and thrombocytopenia in seven and six patients, respectively (Table 4). Myelosuppression is a major toxicity of docetaxel given in a 3-weekly schedule [11]. Irinotecan is also known to be moderately myelotoxic, especially with regard to neutropenia [15]. Therefore, the association of DI with high incidence of grade 3/4 hematologic toxicities in our series was not surprising. Neutropenic fever, which occurred in six (12%) of the 51 cycles, was accompanied by one grade 3 orbital cellulitis and another grade 3 anorectal abscess, but these infections were effectively managed without further morbidity. In addition, no single episode of septicemia happened. Even the five patients who had received previous high-dose chemotherapy and hematopoietic stem cell transplants tolerated DI therapy well. Of the non-hematologic toxicities, grade 2 fluid retention, grade 2/3 neurotoxicity, and grade 3 diarrhea resulted in dose reduction of docetaxel or irinotecan; however, these toxicities were manageable with supportive care alone.

Despite the use of intensified dosage, both docetaxel and irinotecan required one dose-level reduction in two patients for each. Chemotherapy schedule was delayed in only one patient. Moreover, all toxicities were manageable and no treatment-related mortality occurred. These results indicate that DI therapy was relatively safe in our series.

This study had several limitations. First, our cohort size was small, although the patients suffered from uniform pathology, unlike many other trials of pediatric sarcomas with various histologies [8, 10]. Second, docetaxel was administered every 3 weeks instead of weekly in an attempt to reduce cost, which may have resulted in enhanced myelotoxicity.

This study was the first to show the usefulness of the DI combination in patients with ESFT, and is characterized by one of the highest representations of heavily pre-treated patients reported so far. Testing this combination at earlier stage of disease, such as first relapse, could be interesting. Moreover, because of the short remission period with unsatisfactory PFS even in patients who achieve CR, the benefit of adjunct treatments, such as biological agents, in combination with or following DI therapy, warrants evaluation.

## Conclusions

This prospective study suggests that docetaxel in combination with irinotecan shows an encouraging antitumor efficacy with a manageable toxicity profile in children and young adults with recurrent and/or refractory ESFT, who have been heavily pre-treated. However, this finding should be verified in a larger cohort.
